# Memory in astrocytes: a hypothesis

**DOI:** 10.1186/1742-4682-3-2

**Published:** 2006-01-18

**Authors:** Robert M Caudle

**Affiliations:** 1 Department of Oral and Maxillofacial Surgery and Diagnostic Sciences, University of Florida College of Dentistry, Gainesville, Florida 32610, USA; 2 Department of Neuroscience and the McKnight Brain Institute, University of Florida College of Medicine, Gainesville, Florida 32610, USA

## Abstract

**Background:**

Recent work has indicated an increasingly complex role for astrocytes in the central nervous system. Astrocytes are now known to exchange information with neurons at synaptic junctions and to alter the information processing capabilities of the neurons. As an extension of this trend a hypothesis was proposed that astrocytes function to store information. To explore this idea the ion channels in biological membranes were compared to models known as cellular automata. These comparisons were made to test the hypothesis that ion channels in the membranes of astrocytes form a dynamic information storage device.

**Results:**

Two dimensional cellular automata were found to behave similarly to ion channels in a membrane when they function at the boundary between order and chaos. The length of time information is stored in this class of cellular automata is exponentially related to the number of units. Therefore the length of time biological ion channels store information was plotted versus the estimated number of ion channels in the tissue. This analysis indicates that there is an exponential relationship between memory and the number of ion channels. Extrapolation of this relationship to the estimated number of ion channels in the astrocytes of a human brain indicates that memory can be stored in this system for an entire life span. Interestingly, this information is not affixed to any physical structure, but is stored as an organization of the activity of the ion channels. Further analysis of two dimensional cellular automata also demonstrates that these systems have both associative and temporal memory capabilities.

**Conclusion:**

It is concluded that astrocytes may serve as a dynamic information sink for neurons. The memory in the astrocytes is stored by organizing the activity of ion channels and is not associated with a physical location such as a synapse. In order for this form of memory to be of significant duration it is necessary that the ion channels in the astrocyte syncytium be electrically in contact with each other. This function may be served by astrocyte gap junctions and suggests that agents that selectively block these gap junctions should disrupt memory.

## Background

Until recently astrocytes were considered to play no more than a supportive role for neurons in the central nervous system. This view has now been supplanted by a more active participation of astrocytes in information processing, where the astrocytes not only receive and respond to neuronal input, but also transmit signals to neurons [1-9]. These findings indicate that astrocytes contribute to the processing of information. In support of this concept it was recently demonstrated that spinal cord astrocytes are necessary to support hyperalgesia produced by peripheral injury [10-12]. Blocking gap junctions in the astrocytes suppressed hyperalgesia, which suggested that the astrocytes were processing the nociceptive information and regulating the function of spinal cord neurons [10]. These results are similar to work reported by Hertz et al. and Ng et al. who demonstrated that astrocytes are critical for the establishment of learned behaviors [13,14]. Furthermore, recent studies indicate that several general anesthetics suppress the function of astrocyte gap junctions at concentrations that are relevant for loss of consciousness [15,16]. These data suggest that the anesthetic properties of these agents may be mediated at least in part by their actions on astrocytes and may indicate some role for astrocytes in consciousness.

In a recent review Robertson outlined an astrocentric hypothesis of memory [17] as an alternative to the current neurocentric or synaptic based theories. In this hypothesis Robertson concludes that because astrocytes form large syncytium via gap junctions and that they are connected to neurons through synapses these cells can store and "bind" diverse information. In this intriguing review Robertson hypothesizes that information is stored as a result of gap junctional plaques converting to a crystalline configuration that is a closed, high resistance, state of the gap junctions. As a result of these altered gap junctions ion flow between astrocytes is restricted resulting in a functional memory.

In examining the idea that astrocytes might play a major role in information processing it seemed prudent to examine other potential memory mechanisms that could support information processing in astrocytes. In experiments examining electrical potentials and calcium fluxes in astrocytes it was demonstrated that these cells can, on an individual basis, support potentials for several seconds [1,2,6,7]. These data suggest that ion channel activity in a group of gap junction linked astrocytes could retain information for substantial periods of time. Thus, the ion channels mediating the astrocyte potentials could function to store and process information in the central nervous system. This paper examines the possible role of ion channels in storing information in astrocytes.

## Results and discussion

### Similarity of ion channels to cellular automata

**Figure 1:**
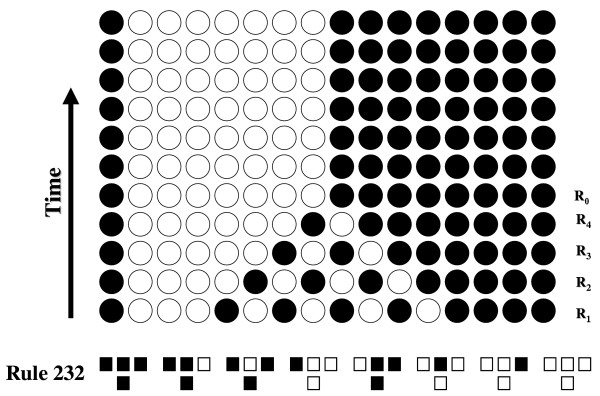
Memory in cellular automata. A sixteen unit one dimensional cellular automaton was constructed using binary units and Wolfram's rule number 232. This rule is illustrated at the bottom of the figure where the three squares on top are the current states of three adjacent units and the single square below is the resultant state of the middle unit during the next iteration. Open squares indicate state 0 and filled squares indicate state 1. The initial representation (R_1_) was generated by randomly setting the state of each unit to either 0 (open) or 1 (filled). The time series was then calculated. Note that the memory of this system extends from R_1 _to R_4 _where the representations change with each iteration. Starting at R_0 _the units no longer change state indicating that all information about R_1 _is lost.

Ion channels communicate with each other via changes in voltage, changes in calcium concentrations or through other second messenger systems. In voltage gated ion channels, for example, the rules governing the relationship between channels specify that if neighboring channels alter the local membrane potential to some threshold the channel under observation will change state, i.e. open or close. Each ion channel functions as an independent unit that monitors information transmitted from its nearest neighbors. As a result of the information processing occurring at the single ion channel level ensembles of ion channels are capable of performing relatively complex functions, such as the generation of action potentials. This form of information processing by ion channels is remarkably similar to models known as cellular automata [18,19]. In cellular automata simple units that are capable of existing in a finite number of states are linked together using rules for the transfer of information between the units. The states occupied by the units and the rules of information transfer determine what state each unit will occupy in the next time period. These models have been extensively studied and demonstrate the emergence of complex behavior [20,21]. Some cellular automata have even demonstrated universal computation [22]. To illustrate how a cellular automata stores and processes information a one dimensional cellular automaton in which the units are binary (they are either in state 0 or state 1) is presented in Figure 1. The rule used was the mean of three units rounded to the nearest integer determines the state of the middle unit in the next iteration. This model was studied at length by Wolfram and this rule is Wolfram's rule number 232 [20,21]. In figure 1 the initiating event (Representation 1 (R_1_)) was produced by randomly setting the states of the units in the automata. The time series was then calculated. In the figure it is evident that from R_1 _to R_4 _the automaton changes representations, but after R_4 _the cellular automaton reaches a steady state and the representations no longer change. This stabile representation is the attractor R_0_. The transition period from R_1 _to R_0 _is the memory of the automaton. At each iteration prior to R_0 _the automaton retains information that can be used to determine something about the initial configuration. However, when the automaton reaches R_0 _all information about the initial configuration has been lost. In astrocytes the ion channels in the membrane are distinct units with a finite number of states and they communicate with each other through a simple set of rules, i.e. a change in voltage or in Ca^2+ ^concentration. Therefore, the astrocytes' membrane ion channels are acting as a two dimensional cellular automaton. As with the automaton presented in figure 1 the initiating event can be inferred based on the configuration of the entire ensemble of ion channels up until the ion channel configuration returns to the attractor representation (R_0_). At this point all information about the initiating event is lost. This concept suggests that ion channels working in collection can store information for at least brief periods of time. The remaining question is the maximum duration of memory in this type of system.

### Memory in cellular automata

**Figure 2:**
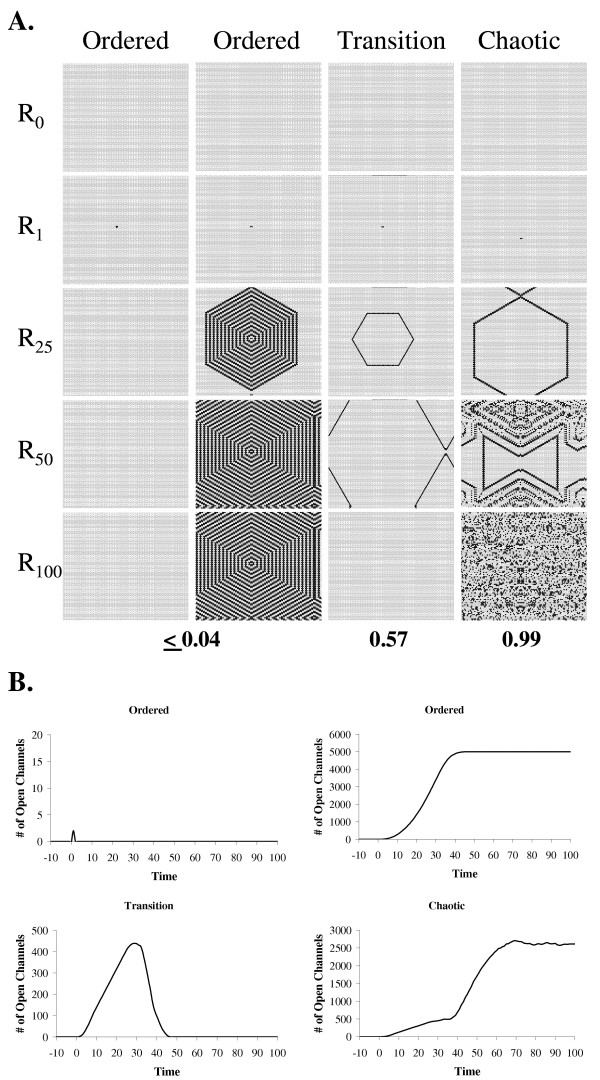
Two dimensional cellular automata operating between order and chaos behave like excitable membranes in biological cells. **A. **A two dimensional cellular automaton was constructed with the program CaSim using units with four states, i.e. one open, one closed and two inactive states. Four different rule sets were used to generate the four time series in the figure. The cellular automaton was seeded at R_1 _by setting two units to the open state and the times series calculated. The configuration of the cellular automata at iterations 0, 1, 25, 50 and 100 are presented in the figure for the four rule sets. The entropy of the rule sets was determined by calculating the probability of each state (P_s_) from 10 runs of 1000 iterations. For these calculations 10 percent of the units were set to the open state at R_1_. Entropy was calculated using the equation: entropy = -∑ P_s _ln (P_s_). The entropy of each rule set was then expressed as a ratio of the calculated entropy to the maximum entropy (bottom of the figure). The maximum entropy is when all four states have a probability of 0.25. **B. **The "potentials" generated by the rule sets in A were graphed by plotting the number of open channels versus time. These plots indicate that only the transition rule set produces channel openings that are similar to action potentials in biological membranes.

In a series of interesting experiments Langton examined the properties of cellular automata that optimize information storage and processing [23]. In these experiments he varied the rules by which the cellular automata operated and measured the resulting chaotic nature of the system. Langton found that automata whose rules made them operate at the junction between ordered and chaotic behavior were able to store information for the longest period of time. Memory dropped off markedly on either side of this phase transition. To illustrate how the chaotic nature of the cellular automata might influence memory a two dimensional cellular automaton with four different rule sets and a Moore neighborhood (8 neighbors) was set up (Figure 2A). The units in the automaton could occupy four different states, i.e. one open, one closed and two inactive. The cellular automaton was seeded with two units in the open state to invoke the initial representation R_1_. The left hand column illustrates a rule set that produces ordered behavior. Note that a signal cannot propagate in this cellular automaton. The second column demonstrates another form of ordered behavior where the behavior immediately becomes repetitive. This cellular automaton, like the one to the left of it, cannot process information due to the inability of the automaton to transition to novel representations. The third column is a rule set that produces behavior at the border between order and chaos. The net result is the smooth propagation of an "action potential" throughout the cellular automaton with the system eventually returning to the attractor representation R_0_. The final column illustrates a chaotic system that evolves rapidly into a random pattern of channel openings. The nearly random behavior prevents proper processing of information since there is no relationship between successive representations. Figure 2B illustrates the "potentials" produced by these different rule sets by plotting the number of open channels versus time. These models demonstrate that only the rule set with behavior at the transition between order and chaos produces a potential that is similar to an action potential observed in biological systems. Note that the rules that produce ordered behavior either returned to the attractor representation R_0 _very rapidly or never returned to R_0_, suggesting that the systems are incapable of supporting information storage. The chaotic rule set also never returns to the attractor, which also indicates that the system cannot retain information for significant periods of time. Only the rule set that produced behavior between order and chaos could retain information about the initial event R_1 _for a period of time and then return to the attractor representation. Based on the similarity of the potentials generated by the transition rule set these models suggest that the ion channels in the membranes of biological cells function as cellular automata with rules that set the behavior at the boundary between order and chaos. This region of the order to chaos spectrum balances information storage with transmission, which, in turn, supports information modification [23].

In addition to examining the length of memory in cellular automata relative to the chaotic nature of the automata, Langton [23] evaluated how the number of units in an automaton influenced memory. In these experiments Langton used rules that produced automata that operated in the order/chaos phase transition and then varied the number of units in the automata. He found that there was a log-linear relationship between the time that the cellular automata stored information and the number of units in the automata. This indicated that the addition of units to the automata exponentially increased the amount of time the automata stored information. This relationship is an extremely powerful property of cellular automata that has evolutionary significance for biological systems that process information with ion channels. The exponential relationship between memory and the number of units in an automaton indicates that a biological system simply has to add more units (ion channels) to its calculating device in order to dramatically increase its memory. With an increase in memory duration the complexity of the calculations that can be performed also increases [23].

### The human cellular automaton

**Figure 3:**
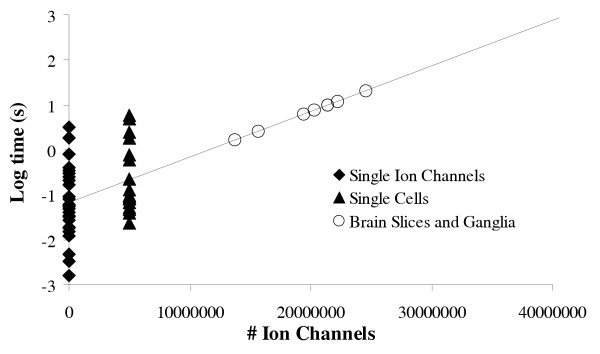
Memory as a function of the number of ion channels. Data was collected from the literature for the open/closed times for single ion channels, the length of potentials evoked in single cells and the length of potentials in groups of cells in brain slices or mollusk ganglia. The logs of the single ion channel and single cell data were graphed versus the number of ion channels. Cells were estimated to have 10^6 ^ion channels. The slope of the line defined by these two points was determined and the length of the potentials in the brain slices and mollusk ganglia were plotted onto the graph.

The findings of Langton indicate that as a cellular automaton is increased in size the duration of memory increases. In the astrocentric hypothesis large numbers of astrocytes are connected through gap junctions [10,17,24-27], which suggests that astrocytes form extensive ion channel cellular automata. To examine the potential memory duration for a human brain sized cellular automaton data was collected from the literature for maximum ion channel open and closed times, duration of potentials evoked in single cells by very brief stimuli and the duration of potentials in brain slices and mollusk ganglia. The recordings in the slices and ganglia used for this analysis represented a large number of cells in the tissue rather than a single cell in the slice or a population response to a single synaptic event. Since data are limited for astrocytes, potentials from all forms of excitable cells were collected. In figure 3 the log maximum length of time reported for single ion channels to transition through an open and closed cycle and the log of the duration of evoked whole cell potentials were plotted versus the number of ion channels. For whole cells the number of ion channels was estimated to be 10^6^. A regression line was fitted to these two sets of data. The duration of potentials from the slices and ganglia were then plotted on this line and the number of ion channels needed to produce these potentials was estimated by extrapolation. These potentials appeared to be generated by 10^7 ^to 10^8 ^ion channels. This finding suggests that Langton's relationship of the number of units to length of time that information is stored in cellular automata holds true for ion channel cellular automata. Note that for convenience there was no attempt to limit the data collected to any one type of ion channel, cell type, or species. The assumption used here is that all biological systems evolved a similar mechanism to process information with ion channels and, as such, their ion channels have similar properties.

To generate an estimate of the total number of ion channels in a human astrocyte cellular automata the number of astrocytes was approximated to be 10^13 ^[28]. With 10^6 ^ion channels/cell this suggests 10^19 ^ion channels in a human cellular automaton. Using the estimate of 10^19 ^ion channels in the human cellular automaton the predicted duration of memory was extrapolated from the slope of the line in figure 3. The relationship between memory and the number of ion channels was estimated to be . Where t is time and N is the number of ion channels in the system. This calculation yielded a predicted maximum memory for a human sized astrocyte cellular automaton of  years. Therefore, for all practical purposes, the predicted maximum duration of memory in human cellular automata is infinite. What is most notable about this memory is that it occurs without fixing the information to any physical structure such as a synapse or cell as predicted in Hebb's postulate [29]. The information is stored as a succession of representations, or ion channel configurations, with each individual representation lasting only a short period of time. The configuration of the ion channels is organized by the incoming information and then as this organization dissipates over time the information is lost. In thermodynamic terms the entropy of the system is decreased by the storage of information and, as the calculation presented above indicates, it takes a substantial amount of time for the entropy to return to baseline levels. Admittedly, the estimates for the number of ion channels and the number of astrocytes that make up a single syncytium are crude; however, even if the estimates are off by several orders of magnitude the overall conclusion that the potential duration of memory in a human ion channel cellular automaton is infinite, from a biological frame of reference, remains valid.

Another interesting comparison to be made between the astrocentric hypothesis and the neurocentric hypothesis is that there are  distinct representations or unique configurations of the ion channels. Using 10^12 ^neurons each possessing 10^3 ^synapses we can estimate that there are 10^15 ^synapses in a human brain [28] and a potential for  distinct representations or unique configurations of the synapses. The term k is the number of states that an individual ion channel or synapse can take. These calculations demonstrate that the potential information processing capacity of the astrocytes using ion channels is many orders of magnitude larger than the capacity of neurons using synapses.

### Associative memory in cellular automata

**Figure 4:**
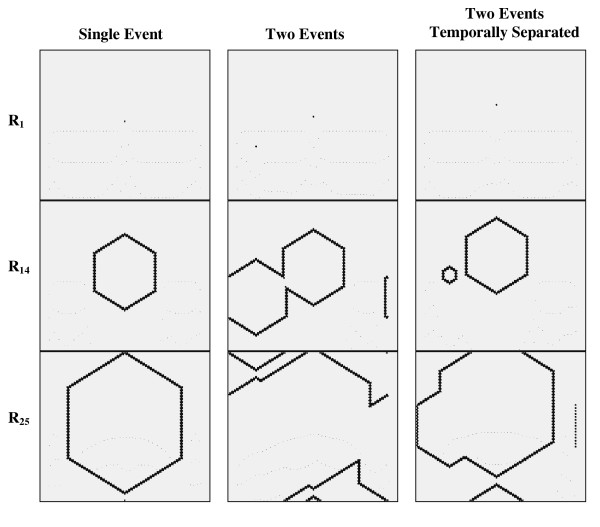
Associative memory in cellular automata. A cellular automaton operating at the transition between order and chaos was setup as described in figure 2 using the program CaSim. Three different stimuli were used. Iterations 1, 14 and 25 are presented in the figure. In the left column the cellular automaton was seeded by setting one unit to the open state at R_1 _(Single Event). In the center column two units were seeded at R_1 _(Two Events). In the right hand column the cellular automaton was seeded by setting one unit to the open state at R_1 _and a separate unit to the open state at R_10 _(Two Events Temporally Separated). Note that each time series generates a different pattern of channel openings (representations) indicating that the two events in the second and third columns have produced unique memories by associating the events. Also note that the difference in representations produced by the automaton in the second and third columns indicates that the cellular automaton stores temporal information about the events. Therefore it is concluded that a two dimensional ion channel cellular automata is capable of associative memory.

An important component of memory is the ability to associate two or more events. In an ion channel cellular automata this is accomplished by the fact that the series of representations produced by a single event is significantly different from that produced by two events. Figure 4 demonstrates the ability of a cellular automaton to associate information from two events. In the first column a single event produces a series of representations as the automaton progresses. In the second column two events occur simultaneously. The two events produce a series of representations that are distinct from the single event presented in the left column. This indicates that the two events have been associated to produce a unique memory.

Another interesting facet of ion channel cellular automata is that because they are dynamic systems they can readily store temporal differences between events. In the right hand column the two events are separated by ten units of time resulting in a series of representations that differs from either the single event in the left hand column or the two simultaneous events in the middle column. These observations suggest that the proposed astrocyte memory system can associate memories and that temporal information can be stored.

### Research supporting astrocyte cellular automata as memory systems

In studies published over forty years ago Hyden demonstrated that glia were critical for memory [30-32]. More recent work using the one-trial aversive learning paradigm in chicks has confirmed Hyden's findings [13,14,33]. In these studies inhibitors of astrocyte function were found to block both short term and intermediate term memory, but, when administered later, had no effect on the long term retention of the learned behavior. During the short and intermediate periods it was demonstrated that ion fluxes in astrocytes are critical [13,33,34] for memory suggesting that the astrocyte ion channels may store information in the chicks for a brief period of time, approximately 60 minutes, while the appropriate rewiring of the neuronal circuitry takes place. It is important to note that this behavioral model involves both memory and learning, while the cellular automata hypothesis presented here is related purely to memory. Memory is the ability of an organism to store information about events in a retrievable format, whereas learning involves a change in behavior or potential behavior. Thus, a consolidated learned behavior, as occurs in the one-trial aversive learning paradigm, is likely to be the result of neuronal rewiring. Furthermore, it does not require the organism to retain any specific memory of the event that precipitated the change in behavior beyond the length of time necessary to produce the rewiring. In this light, the chick in the aversive learning paradigm may actually recall the aversive stimulus for the short and intermediate term memory periods, which require astrocytes, but may not retain any recollection of the event once the aversive behavior has been established. It is enough for the chick to avoid certain objects without remembering why it needs to avoid them. The distinction between memory and learning is important because the two processes are likely mediated by different mechanisms. In the current hypothesis the ion channel cellular automata would be responsible for the specific memory of the event while changes in synaptic strength of the neurons would be responsible for learning and maintaining the new behavior. Astrocyte memory could support learning, but learning does not necessarily support the memory of events.

In addition to proposing that glia were involved in memory, Hyden predicted that mental diseases may involve glia [35] as reported in [34]). In the ion channel cellular automata hypothesis it is critical that the ion channels operate at the junction between order and chaos. Departure from this behavior is predicted to produce pathology. Deviation to the ordered side of the spectrum might produce depressive types of behaviors in the organism and memory deficits while deviation to the chaotic side might produce psychotic or manic types of behaviors that are also associated with memory deficits. Several studies have demonstrated that long term treatment with antidepressant drugs at clinically relevant doses alters protein expression and function in astrocytes [36-41] and long term treatment with lithium ion results in suppression of mRNA for sodium-dependent inositol transporter in astrocytes [34]. The length of treatment required for the change in astrocyte proteins is consistent with the onset of the therapeutic effect of these agents. These studies suggest that these psychoactive agents may adjust the activity of astrocyte ion channel cellular automata toward the order/chaos border, thus improving the function of the memory system. Therefore, a number of studies, spanning over forty years, indicate that astrocytes are important for memory and possibly for the therapeutic effect of psychoactive drugs, which is consistent with the astrocyte ion channel cellular automata hypothesis.

## Conclusion

In this study the hypothesis that astrocytes could store information in the central nervous system was considered. Based on the similarity of membrane ion channels to mathematical models known as cellular automata it seems reasonable to conclude that ion channels in astrocytes could store information for significant periods of time. This storage system does not rely on physically fixing information to any structure such as a synapse; rather information is stored by organizing the activity of the ion channels. If this concept is correct it suggests that neurons may use astrocytes as a dynamic information sink. In theory, this information would remain readily available to the neurons for extended periods of time. Furthermore, this hypothesis indicates that to store information for significant periods of time the ion channels in the astrocyte syncytium must be in electrical contact with each other. This function could be served by the astrocytes' gap junctions. Thus, we can predict that agents that selectively block astrocyte gap junctions should disrupt memory. Clearly, further work is needed to verify this theoretical framework for memory in nervous systems.

## Methods

### One dimensional cellular automaton

A 16 unit one dimensional cellular automaton was set up with each unit having 2 states. The rule used for this automaton was Wolfram's rule number 232 [20,21]. In this rule each unit is updated by averaging the states of the unit with its two nearest neighbors and then rounding to the nearest integer. The time series for this cellular automaton was calculated by hand.

### Two dimensional cellular automata

To examine the effects of different rule sets on 2 dimensional cellular automata the program CaSim [42] was used. A matrix of 100 × 100 units with a Moore neighborhood (eight neighbors) was set up with various rules. Each unit had 4 states. The entropy of the different rule sets was calculated using the equation entropy = -∑ P_s _ln (P_s_), where P_s _is the probability of a unit occupying a particular state. The probabilities of the different states were determined from 10 runs of 1000 iterations for each cellular automaton. For these calculations the cellular automaton was seeded for each run by randomly setting ten percent of the units to the open state. The maximum entropy was calculated using the probability of 0.25 for each of the four states. The ratio of the calculated entropy of the rule set to the maximum possible entropy was used as an indicator of the chaotic nature of the system. Thus an entropy ratio of 0 is a completely ordered rule set and a ratio of 1 is a completely chaotic rule set.

For the examples presented in the figures the cellular automata where seeded with either 1 or 2 units set to the open state.

### Duration of memory versus the number of ion channels

To calculate the relationship between the number of ion channels in a system and the duration of information storage by the ion channels data was collected from published sources. The maximum open and closed times for various ion channels were obtained [43-54] and the open to closed cycle was used as the duration of memory in single ion channels. Similarly, potentials recorded in single cells were obtained [55-62] and used as an indication of the activity of multiple ion channels in concert. The log of the values for the duration of the responses in the ion channels and cells were plotted versus the number of ion channels. The number of ion channels in the cells was estimated to be 10^6^. A line was then fitted to the two points and the log of the duration of potentials in slices and ganglia [63-71] were plotted on the line.

## Competing interests

The author(s) declare that they have no competing interests.
